# Noise Improves Visual Motion Discrimination via a Stochastic Resonance-Like Phenomenon

**DOI:** 10.3389/fnhum.2016.00572

**Published:** 2016-11-23

**Authors:** Mario Treviño, Braniff De la Torre-Valdovinos, Elias Manjarrez

**Affiliations:** ^1^Instituto de Neurociencias, Universidad de GuadalajaraGuadalajara, México; ^2^Centro Universitario de Ciencias Exactas e Ingenierías, Universidad de GuadalajaraGuadalajara, México; ^3^Instituto de Fisiología, Benemérita Universidad Autónoma de PueblaPuebla, México

**Keywords:** decision making, discrimination, visual perception, visual cortex, humans, stochastic resonance

## Abstract

The stochastic resonance (SR) is a phenomenon in which adding a moderate amount of noise can improve the signal-to-noise ratio and performance of non-linear systems. SR occurs in all sensory modalities including the visual system in which noise can enhance contrast detection sensitivity and the perception of ambiguous figures embedded in static scenes. Here, we explored how adding background white pixel-noise to a random dot motion (RDM) stimulus produced changes in visual motion discrimination in healthy human adults. We found that, although the average reaction times (RTs) remained constant, an intermediate level of noise improved the subjects’ ability to discriminate motion direction in the RDM task. The psychophysical responses followed an inverted U-like function of the input noise, whereas the incorrect responses with short RTs did not exhibit such modulation by external noise. Moreover, by applying stimulus and noisy signals to different eyes, we found that the SR phenomenon occurred presumably in the primary visual cortex, where these two signals first converge. Our results suggest that a SR-like phenomenon mediates the improvement of visual motion perception in the RDM task.

## Introduction

Visual motion perception is crucial for survival because it allows a rapid estimation of the speed and direction of relevant moving objects in visual scenes. A widely used method to measure visual motion perception in monkeys and humans is to combine a random dot motion (RDM) stimulus (Newsome and Pare, 1988) with a psychophysical test based on two response options (Ward et al., 2002; Gold and Shadlen, 2007). In such a task, the subjects visualize an array of dots some of which move coherently in one direction, whereas the rest moves in random directions. The subjects must carefully observe the moving dots, choose between two main possible directions of motion and report their choice with a motor response such as doing a saccade or pressing a button with their index finger. There is ample experimental evidence that suggests that such ongoing projection of the dots moving together in the same direction (i.e., the coherence of motion) provides cumulative evidence over time to inform the decision (Shadlen and Newsome, 2001; Gold and Shadlen, 2007). Indeed, many decision theories suggest that animals make their choices by accumulating sufficient stimulus information until they emit their responses (Smith and Ratcliff, 2004; Gold and Shadlen, 2007; Treviño et al., 2013). The build-up of such discriminative information is sensitive to relevant parameters such as the intensity and quality of the stimulus, prior response probabilities, and reward probabilities (Smith and Ratcliff, 2004; Treviño et al., 2013; Treviño, 2014; Herrera and Treviño, 2015). However, additional, relevant, and not yet explored variables could also interact and influence behavioral output in the RDM task. For instance, an external noise source could have a strong impact on the choice performance of the subjects by degrading the quality of discriminative information, a perturbation which could be exploited to investigate the core mechanisms involved in optimal decision making.

Intuitively, one would expect that excessive random noise would interfere with the build-up of discriminative information leading to worse detection performance, degraded estimation accuracy, and reduced generalization capacity (Moss et al., 2004). What would be the effect of using smaller noise sources? Recent studies have revealed that low amplitude noise can play a ‘constructive’ role in the detection of weak signals through a mechanism called stochastic resonance (SR; Moss et al., 2004; McDonnell and Abbott, 2009; McDonnell and Ward, 2011). SR refers to a phenomenon where adding an optimal level of white noise can increase the signal-to-noise ratio (SNR) in non-linear systems, thereby improving the detection of weak stimuli (Ward et al., 2002; Moss et al., 2004; McDonnell and Abbott, 2009; McDonnell and Ward, 2011). Although, originally described in a climate change study (Benzi et al., 1981), the SR phenomenon has also been reported in sensory, motor, and sensorimotor systems in humans (Simonotto et al., 1997; Leopold et al., 2002; Moss et al., 2004; Sasaki et al., 2006; Martinez et al., 2007; McDonnell and Ward, 2011; Medina et al., 2012; Mendez-Balbuena et al., 2012). A general property of SR is that the response of the system under investigation vs. the input noise renders an inverted U-shaped SNR curve with a peak response increment at an intermediate noise level. It is precisely at this ‘optimal’ noise value at which SR increases the system’s sensitivity to detect information-carrying signals. SR is a phenomenon that also has been studied in models of single neurons (Bulsara et al., 1991; Wiesenfeld et al., 1994; Lee and Kim, 1999) and in models of small-world neural networks (Perc, 2007; Ozer et al., 2009; Wang et al., 2009; Yilmaz et al., 2013) due to the fact that it’s influenced by the network topology. The study of SR in neural networks could be of particular importance for the interpretation and discussion of psychophysical experiments involving the application of external noise.

Stochastic resonance has been shown to enhance human visual perception during the addition of pixel-noise to static scenes (Simonotto et al., 1997; Moss et al., 2004). Indeed, visual noise can increase visual contrast detection sensitivity around the threshold level, and thereby improve relevant processes such as pattern recognition and the perception of ambiguous and 3-D figures (Leopold et al., 2002; Sasaki et al., 2006). However, despite all these evidence showing the contribution of SR in visual processing, the relationship between external visual noise and visual motion discrimination is still unexplored. Here, we investigated how adding a dynamic source of background pixel-noise influenced the ability of subjects to perceive motion direction in the RDM task. We found an improvement in the steady-state discrimination of the direction of low-coherence movement by the addition of white background noise with low luminance. Indicative of an SR-like phenomenon, the psychophysical responses of the subjects followed an inverted U-like function of the luminance of the input noise. Notably, this response pattern was absent in error trials with small reaction times (RTs), suggesting that the SR-phenomenon operated at a perceptual level. We confirmed that the SR occurred both in experiments in which the volunteers controlled their decision time autonomously and also when the experimenter fixed the stimulus presentation times. The overall repeatability and stability of our results suggest that the SR might play a major role in the way the human visual system processes dynamic sensory stimuli. The SR phenomenon could be employed to refine the processing of dynamic visual stimuli enhancing human performance in non-invasive ways.

## Materials and Methods

### Participants

We performed the experiments with 149 healthy subjects (62 men and 87 women). Their mean age was 23 ± 1 years (a minimum of 17, a maximum of 63, mode of 19). Most participants were right-handed (>88%), with normal or corrected vision, and with no record of auditory, tactile, visual and motor disorders; also without detectable neurological disorders or history of drug abuse. The participants had different educational degrees (elementary school, *n* = 7; secondary school, *n* = 5; high-school, *n* = 64; college degree, *n* = 46; master’s degree, *n* = 16; doctorate degree, *n* = 7; post-doctorate, *n* = 4). We performed the experiments with the informed consent of each participant and with the approval of the ethics committee of the Instituto de Neurociencias, Universidad de Guadalajara. Our procedures conformed to the Declaration of Helsinki (1964) established by the World Medical Association. All subjects were naïve to the tasks described in the following section.

### Motion Discrimination Task

For motion psychophysics, we employed a two-alternative, forced-choice, RDM direction discrimination task (Newsome and Pare, 1988; Newsome et al., 1989; Shadlen and Newsome, 2001; Gold and Shadlen, 2003). We programmed the RDM task using the psych-toolbox (Brainard, 1997; Pelli, 1997) for Matlab (TheMathWorks, Inc.; Natick, MA, USA) running on Windows 7 (Microsoft) installed on two standard desktop machines (Dell Precision T3610 Tower Workstation; AMD FirePro W7000). All subjects received written instructions of the task and performed a brief training phase which consisted of obtaining 20 correct consecutive trials. During the training phase, we used auditory tones with pure frequencies to indicate right and wrong choices. We performed all experiments in a silent room.

**FIGURE 1:**
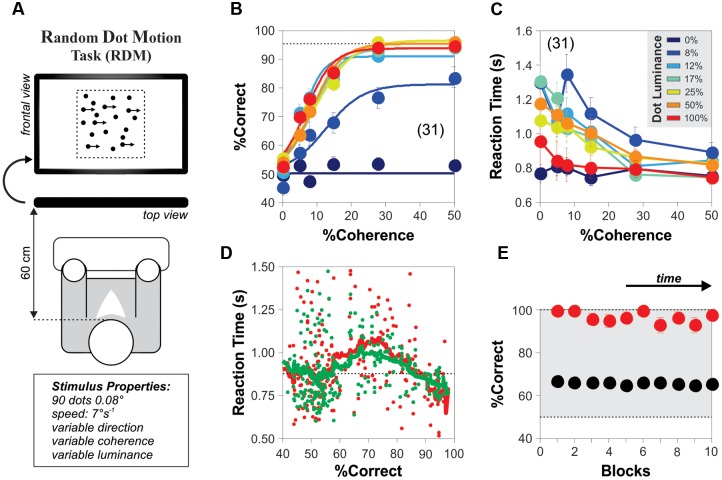
**Steady-state psychometric curves for the random dot motion (RDM) task.** **(A)** Cartoon of the RDM task with an array of 90 moving white dots presented at the center of a black computer screen. The dots appear in black for visualization purposes only. A percentage of the dots moves in the same horizontal direction (i.e., the percentage of coherence) whereas the rest moves with random directions. The (left of right) direction of the coherent movement varies pseudo-randomly over consecutive trials. We manipulated the difficulty of the task by varying the proportion of coherently moving dots and their luminance (i.e., stimulus luminance). **(B)** Group-averaged psychometric curves (20 repetitions per condition) depicting the %Correct choices as a function of motion coherence (x-axis), with increasing levels of stimulus luminance represented in color (from blue to red: 0, 8, 12, 17, 25, 50, 100%; category intervals based on a logarithmic scale). The curves saturate with stimulus luminance > 12%. For trials with 0% coherence, the y-axis label represents the % left choices. **(C)** Group-averaged reaction times (RTs) from the choices shown in **(B)**. Inset: color code for stimulus luminance (same code for previous panel). **(D)** Plot of the average RT (correct choices in green; incorrect choices in red) vs. %correct choices (i.e., the probability of making a correct choice) for single-subjects solving the task with different combinations of coherence and stimulus luminance. Responses are faster in easy and difficult conditions but slower in intermediate conditions. **(E)** Correct choices using a stimulus intensity of 100% (red dots) or by taking the average of all stimulus luminances (black dots) remain stable throughout the duration of the experiments (10 blocks of 200 trials each). Number of subjects in parenthesis.

Each subject performed two blocks of 1000 trials each with a 20 min break between blocks. **Figure 1A** illustrates the basic paradigm for measuring direction-discrimination consisting on a stimulus of moving dots projected on a black screen on a computer monitor (1024 × 768 pixels @ 60 Hz; viewing distance: 60 cm; we inverted the polarity of black and white colors in **Figure 1** to facilitate visualization). The RDM display was black and consisted of a total of 90 white dots (dot size: 2 × 2 pixels = 0.08°) contained within an imaginary square of 13.68° per side placed at the center of the screen, and covering a projection area of 187 deg^2^. The dots moved at 7° s^-1^, a certain percentage of them with a coherent direction to either the left or right while the remainder moved with random directions. We initialized the dots with coherent or randomized directions. The dots had limited lifetimes because when they reached the edges of the projection area, they were randomly repositioned along the dimension (x- or y-axis) that was exceeded (yet keeping their trajectories constant). We changed the direction of coherent movement pseudo-randomly on every trial (Treviño, 2014; Herrera and Treviño, 2015). We controlled the strength of motion in the RDM task by changing the relative proportion of dots moving coherently from 0 to 50%. In some trials, we included the presence of additive background white noise (intensities following a uniform distribution, noise size: 1 × 1 pixel = 0.04°), which was refreshed every frame, and had a user-controlled mean luminance (in %). We manipulated the detectability and interference of the RDM stimulus by varying the luminance of the moving dots from 0 to 100% (Pilly and Seitz, 2009; Carandini and Heeger, 2012), and the luminance of the background noise between 0 and 50%, respectively. We optimized our method to sample coherence and luminance by using category intervals based on a logarithmic scale (Gold and Shadlen, 2003; Kiani and Shadlen, 2009).

We used a ‘double receptor design’ in the set of experiments illustrated in **Figure 4A**. We employed two identical displays separated by a black divider that blocked the line of vision between the eyes (Carmel et al., 2010). With this approach, we explored how the background noise influenced the RDM stimulus at the peripheral visual system (Mori and Kai, 2002; Kitajo et al., 2003).

### Analysis of Discrimination Performance

We recorded the choices and RTs of the subjects on every trial. To indicate their choices, they had to press the ‘M’ button on the keyboard with their right index finger or the ‘C’ button with their left index finger when they perceived the movement to the right or left, respectively (maximum allowed RT of 4 s). We report the %correct responses as the percentage of trials with a particular combination of coherence, dot and noise luminances for which a subject identified the direction of movement in the task. We calculated a %correct choice index (%CCI) by dividing the %correct responses in the presence of noise by the %correct responses with zero noise (ZN; paired trials, see below). We interleaved all ‘noise trials’ with trials with ZN and permuted their temporal order so that the subjects could not predict the testing conditions (Pelli, 1985). This arrangement ensured that the estimation of the %CCI index was insensitive to potential variations in attention during the task because, if existent, those variations would be distributed homogeneously into trials with and without the presence of background noise, from which we extracted the index itself. We varied coherence of the dots, and the luminance of dots and background noise across trials in a pseudo-randomized fashion.

We fitted the %CCI data from individual subjects to two descriptive models (illustrated in **Figure 5**). Our exclusive aim of making such curve adjustments was to extract and compare individual measures linked to the SR phenomenon across subjects. The first model corresponded to an adaptation of a general SNR curve as a function of noise intensity (*l_i_*), as follows:

$$SNR=2.5\log_{10}\left(\frac{2w_{n}\Delta_{0}^{2}A^{2}}{\sqrt{{3\left(g(l_{i}-l^{*})/10\right)}^{4}}}\right)\exp\left(\frac{-\Delta_{0}^{2}}{{2\left(g(l_{i}-l^{*})/10\right)}^{2}}\right)$$

where *A* represents the amplitude of a periodic signal, *w*_n_ is the upper cutoff frequency of the noise, Δ_0_ is the difference between the mean of the subthreshold stimulus and the threshold, *g* is a gain variable, and *l*^∗^ is a translation constant (Moss et al., 2004). In the second model, we approximated the SNR function by adding a logistic curve to a Gaussian distribution, as follows:

$$SNR=\{\frac{L}{1+e^{k\left(c_{i}-c^{*}\right)}}\}+\left\{Amp\cdot \exp\left(\frac{-{\left(c_{i}-\mu \right)}^{2}}{{2\sigma }^{2}}\right)\right\}$$

Where *L* is the maximum value of the logistic function, *k* is the slope of the curve, *c*^∗^ is the *x*-value of the sigmoid’s midpoint, *Amp* is the amplitude of the Gaussian with a Δ mean and Δ standard deviation. We employed standard non-linear programming techniques to fit the experimental data to both of these models (Treviño, 2015).

### Statistical Analysis

We report and illustrate all results as averages ± SEM. We used parametric and non-parametric tests with significance set at *P* < 0.05 for statistical comparisons.

## Results

### Psychometric Curves with the Motion Task

We established the conditions to quantify motion discrimination performance in adult humans. We employed a RDM task originally introduced by Newsome and Pare (1988; see Materials and Methods). It consisted on projecting 90 white moving dots on a black screen and requesting the experimental subjects to report their perceived direction of overall movement when a fraction of the dots moved coherently to the left or right, while the remainder of the dots moved in random directions (**Figure 1A**). We manipulated the task difficulty by adjusting the percentage of coherently moving dots (Gold and Shadlen, 2007) and plotted the averaged psychometric curves as a function of stimulus coherence (x-axis) and luminance (colored lines in **Figures 1B,C**; legend on the inset of **Figure 1C**). The psychometric curves of the subjects had an average peak performance of 91.93 ± 2.50%, a midpoint of 8.65% ± 1.05% luminance and a slope of 23.78 ± 2.52 (%c/%luminance). The peak performance of these response curves had a marked sensitivity to gradients in the luminance of the stimulus at or below 12% (blue line in **Figure 1B**).

We conducted these initial experiments under a ‘free response’ paradigm: we instructed the subjects to discriminate motion direction as best as they could and allowed them to control their decision time autonomously (Treviño et al., 2012, 2013; Treviño, 2014). Under this condition, accuracy rates and RTs vary as a function of task conditions, leading to predictable changes in the probabilities of these two output parameters (Smith and Ratcliff, 2004; Treviño et al., 2013). For example, a shift from speed to accuracy instructions can result in slower yet more accurate responses, whereas the opposite instructions usually decrease the choice accuracy but involve shorter sampling intervals (‘speed-accuracy tradeoff’). We found that the averaged RTs exhibited a monotonic decrease linked to an increase in the stimulus coherence and luminance (**Figure 1C**). Indeed, the mean RTs dropped with increasing coherence from 5 to 50% (RT with 5% coherence: 1.01 ± 0.05 s; RT with 50% coherence: 0.80 ± 0.02 s; Kruskal–Wallis test, *F*_1,12_ = 6.20, *P* < 0.001). This result reflects that the rate of accumulation of discriminative information depends on the difficulty of the task (Gold and Shadlen, 2007; Treviño et al., 2013). Hence, decreasing stimulus discriminability increased the error rates, delaying and dispersing the RT distributions (Smith and Ratcliff, 2004).

We wondered how the averaged RTs (extracted from those randomized trials with identical coherence and luminance levels) related to the probability of making correct choices under conditions with variable difficulty. We plotted the averaged RTs derived from correct (green dots) and incorrect (red dots) choices as a function of the probability of making a correct choice (‘%Correct,’ i.e., the number of correct choices divided by the total number of trials for that combination of stimulus attributes; **Figure 1D**). This plot revealed an inverted bell-shaped pattern of RTs vs. %correct choices, with smaller averaged RTs associated with ‘easy’ and ‘difficult’ task conditions (Smith and Ratcliff, 2004). The peaks of both of these bell-shaped curves occurred at 71% of correct choices and had a 12–20% increase in the averaged RTs compared to those obtained when choosing randomly (i.e., at chance level). We also confirmed that the subjects made all their choices in steady-state throughout the course of the experiment (repeated measures ANOVA test, *P* > 0.5; red dots: stimulus coherence = 50%, stimulus luminance = 100%; black dots: an average of all conditions; **Figure 1E**). Using this task, we were able to indirectly control the steady-state response accuracy of the subjects by simply adjusting the coherence levels of the RDM stimulus.

### Additive Background Noise Increases Visual Motion Perception

**FIGURE 2:**
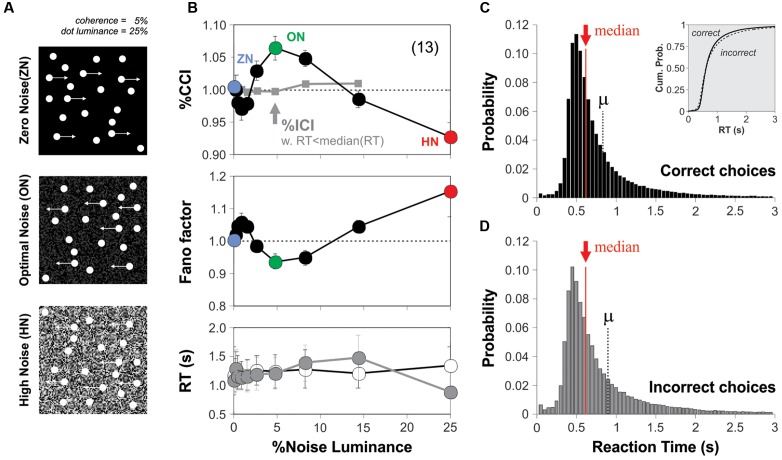
**Background noise enhances motion detection in the RDM task.** **(A)** Cartoon of the RDM task with different background noise average luminance levels [zero noise (ZN): 0% luminance; optimal noise (ON): 5% luminance; high noise (HN): 25% luminance]. The noise source was dynamic; it was refreshed every frame and was added as a background behind the RDM dots. **(B)** Group-averaged %correct choice index (upper panel; %CCI = %correct choices with noise/% correct choices without noise; paired trials, see Materials and Methods) against the luminance of background noise (coherence of 5%; stimulus luminance of 25%). No modulation in %incorrect choice index (%ICI) for incorrect choices that had RTs lower than the median RT (i.e., median split; gray squares in **B**). The Fano factor of the %CCI data presents a local minimum with optimal background noise (green dot; middle panel). The increase in %CCI around 5% of noise luminance (green dot in the upper panel) cannot be explained by changes in the averaged RTs of the choices made in the presence (gray circles) vs. the absence (empty circles) of background noise (middle panel). The skeweed RT distributions for correct **(C)** and incorrect **(D)** choices had similar shapes, means and medians. Number of subjects in parenthesis.

To explore how external visual noise interacted with visual motion perception, we developed the computational tools that allowed us to combine dynamic background pixel-noise (refreshed every frame) with the standard and widely used RDM task (Newsome and Pare, 1988; **Figure 2A**; see Materials and Methods). We asked how adding such noise with different luminance levels influenced the choice performance of naïve subjects solving the RDM task. We tested 13 new participants in conditions where the visual stimulus barely produced a perception of global motion (coherence = 5%; luminance = 25%; correct choices: 63.20 ± 2.48%, one way ANOVA test, *P* = 0.03; **Figure 1B**; Newsome et al., 1989). After performing the experiments, we calculated the group averaged %correct choice index (%CCI) as a function of the luminance of the additive background noise. We took the %CCI index as the amount of %correct choices obtained in the presence of noise divided by those obtained in its absence. The group averaged %CCI revealed a bell-shaped distribution with a peak sensitivity of 6.5 ± 1.8% at a moderate noise luminance of 5% (paired *t*-test, *P* < 0.001; **Figure 2B**; green dot, upper panel). This response pattern resembled a SR-like phenomenon, and the peak value in the %CCI function was consistent with previous SR observations (Zeng et al., 2000; Ward et al., 2002). Regarding the stimulus presentation sequences, we paired the test trials for each specific noise luminance condition with trials lacking noise (see Materials and Methods). This arrangement ensured that the task provided no temporal information about the stimulus (Pelli, 1985), and allowed us to estimate the %CCI independently of possible variations in choice performance due to attentional shifts (or any other factors; Smith and Ratcliff, 2004; Treviño, 2014). We used 100 repetitions per noise luminance.

Because the performance solving the RDM task was highly variable across subjects, we quantified the group response variability by using an index of dispersion known as the Fano factor (the variance of the %CCI values extracted from single subjects divided by their mean). The Fano factor is a normalized measure of the dispersion of a distribution and is useful to capture the degree of randomness of a given phenomenon, as it quantifies how clustered or dispersed are a set of observations. This analysis revealed that the luminance of the visual noise strongly modulated the Fano factor of the %CCI in the RDM task. Indeed, the index was close to 1 when using ZN, yet it dropped and had a local minimum with a background luminance of 5% (under-dispersed values) and then increased above 1 for larger background luminance levels (over-dispersed values). The reduction in the Fano factor was maximum around the same background luminance noise value that elicited the %CCI peak (**Figure 2B**; middle panel). Moreover, as an increase in RTs could explain the improved choice performance (Smith and Ratcliff, 2004), we compared the averaged RTs from trials performed under different noise luminances but found no differences across them (**Figure 2B**; lower panel; paired *t*-test, *P* > 0.05 for all cases).

We next wondered whether this SR-like phenomenon in motion discrimination operated at a perceptual level. We reasoned that if a small amount of *perceived* visual noise is capable of improving the subjects’ performance in the RDM task, then this effect should be absent in those trials that led to incorrect choices. Such lack of effect should be particularly evident in those error trials with smallest RTs, when the subjects made their choices at random (Smith and Ratcliff, 2004; Treviño, 2014). We calculated the %incorrect choice index (%ICI) from error trials produced in the presence and absence of background noise, but only from those that had RTs below the median. Confirming our hypothesis, we found that the %ICI extracted from these error trials was insensitive to the external noise (gray squares in **Figure 2B**, upper panel). Notably, the frequency distributions of RTs for correct and incorrect choices had similar shapes (**Figures 2C,D**). Therefore, we can discard possible misrepresentations of the averaged RTs due to their skewed distributions because (i) the number of trials involved in calculating the %errors in the presence and absence of noise was practically identical, and (ii) both distributions exhibited similarly skewed distributions before the computation of %CCI and %ICI indexes (Whelan, 2008). Altogether, these results demonstrate that an intermediate amount of background noise can increase the %CCI but not the %ICI in the RDM task via a SR-like phenomenon.

### Noise Improves Motion Discrimination in Two Other Task Variants

**FIGURE 3:**
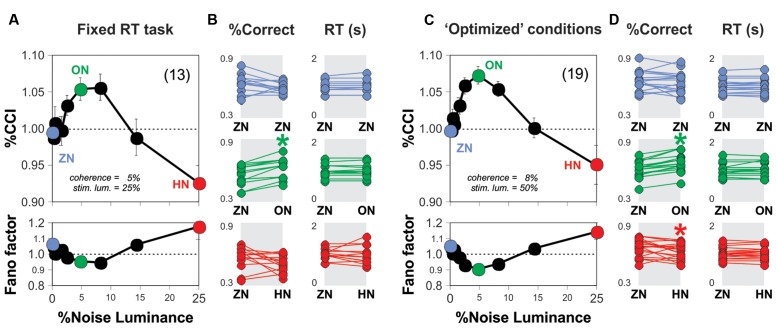
**Optimal background noise increases the %CCI in the RDM task with fixed sensory sampling periods.** **(A)** Group average %CCI (upper panel) and Fano factor (lower panel) vs. background noise luminance using a fixed sampling period of 1 s. **(C)** Experimental results obtained with ‘optimized’ RDM conditions (i.e., 8% motion coherence; 50% stimulus luminance). Dot plots in **(B,D)** illustrate the comparison for %correct choices (left column) and RTs (right column) made in the absence (left) or presence of noise (right side of each panel) per subject (i.e., the dots connected by lines represent individuals). Asterisks depict significant differences assessed with a paired *t*-test. Number of subjects in parenthesis.

We explored whether the SR phenomenon was reproducible across two new variants of the RDM task. The first variant consisted in fixing the stimulus viewing interval to 1 s (i.e., the test trials involved a whole second of stimulus projection, w. coherence = 5% and luminance = 25%). With this setting, we sought to narrow down the variance in the RT distributions, and thereby reduce the possibility that RTs and %CCI were correlated. We conducted experiments in new subjects and obtained a similar bell-shaped %CCI curve with a maximum increase of 5.5 ± 1.5% at a noise luminance of 5% (*n* = 13 subjects; paired *t*-test, *P* < 0.001; **Figure 3A**). Next, we explored whether and how the noise affected the RTs from subjects. Many data distributions can lead to similar appearances when represented with bar-plots, which further invalidates their usage for small datasets. We therefore decided to illustrate the choice and RT data by using scatter plots with the reference (i.e., ZN), on the left, and noise condition, on the right, connected with lines representing each subject (**Figures 3B,D**). In these plots, we can visualize the variability in the performance with the ZN condition across subjects (i.e., each dot represents information from a single subject). Using a paired *t*-test, we confirmed the lack of change in the RT distributions for all levels of noise luminance tested (*P* > 0.05 for all cases; **Figure 3B**). Therefore, the improvement in the %CCI of the RDM task produced by adding background noise cannot be explained by increased sampling intervals.

For the second variant of the task, we first tested four additional stimulus coherence values (two above, and two below 5% coherence) and four additional stimulus luminance levels (two above, and two below 25% luminance). We found that switching the stimulus coherence from 5 to 8% boosted the %CCI by about 4.5 ± 0.3% (Kruskal–Wallis test *F*_4,494_ = 392.38, *P* < 0.001; 25 subjects bootstrapped to 100 cases; not illustrated) whereas using a 50% stimulus luminance increased the %CCI by 2.9 ± 0.3% (against a stimulus luminance of 25%; Kruskal–Wallis test *F*_4,494_ = 253.56, *P* < 0.001; 25 subjects bootstrapped to 100 cases; not illustrated). Taking this information into account, we conducted a new round of experiments on 19 naïve subjects and characterized their %CCI curves when solving the RDM task with 8% of motion coherence and a stimulus luminance of 50% (i.e., ‘optimized’ stimulus parameters; **Figure 3C**). The corresponding averaged %CCI curve had a peak of 7.4 ± 1.2% at a noise luminance of 5%, but a decrease of 4.9 ± 2.6% with 25% noise luminance (paired *t*-test, *P* < 0.0001). There was no change in averaged RTs for all levels of noise luminance tested (paired *t*-test, *P* > 0.05; **Figure 3D**). Remarkably, the %CCI peaks for open RT (**Figure 2B**), fixed RT (**Figure 3A**) and ‘optimized conditions’ (**Figure 3C**) were quite similar to each other (Kruskal–Wallis *F*_2,41_ = 1.33, *P* = 0.51). These results demonstrate that the SR-like phenomenon in the RDM task is reproducible across different experimental settings. It is likely that the %CCI peaks were already saturated at 5% luminance noise in the three conditions tested.

### The Enhancement in Motion Discrimination by Noise Occurs Centrally

**FIGURE 4:**
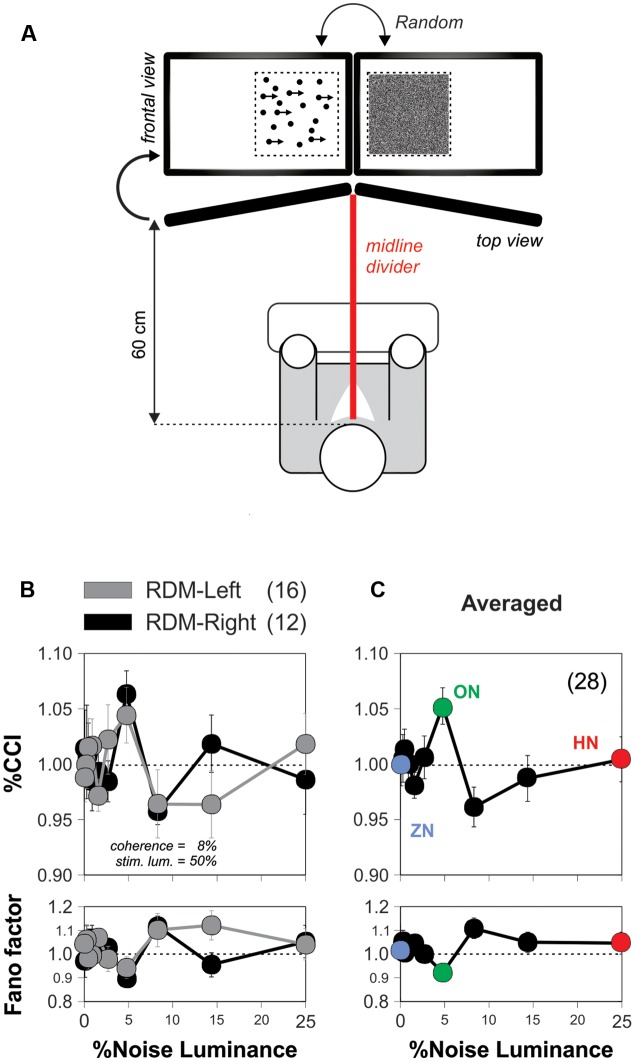
**The discrimination-enhanced-by-noise effect in the RDM task occurs centrally.** **(A)** Diagram depicting the montage of the apparatus for conducting the ‘double-receptor design’ experiments (see Materials and Methods) with two monitors separated by a black divider centered at the midline of the viewing field of the subjects (85° from the midline; RDM and noise displays projected on the sides of the midline divider). Here, we applied the noisy signal to one eye and the stimulus signal to the other eye (w. coherence = 5%; luminance = 25%). **(B)** Average %CCI curves as a function of noise luminance for subjects receiving the RDM stimulus signal to either the left (*n* = 16) or right (*n* = 12) eyes, whereas the visual noise was applied to the opposite eye. **(C)** Average of %CCI curve for both eyes reveals that the SR occurred in the RDM task at a stage where binocular inputs interacted.

In the previous experiments, we presented noise and stimulus signals identically to both eyes. This arrangement is commonly referred to as a ‘single receptor design.’ Under these conditions, the noise and signal interact in the retina and throughout the entire peripheral visual system. A possible explanation for the SR improvement in visual motion discrimination is that the noise enhances the peripheral receptors sensitivity (Mendez-Balbuena et al., 2012), increasing the amplitude of the signal which then percolates to the entire visual system (Moss et al., 2004; Flores et al., 2016), and improves sensorimotor integration (Rodriguez et al., 1999). An alternative, however, could be that this form of SR takes place at a non-retinal location. Interestingly, studies on humans have shown that some forms of visual perceptual learning can be detected by the confluence of inputs from separate eyes (Ball and Sekuler, 1987; Gilbert et al., 2001). These empirical observations motivated us to explore the anatomical locus of the SR-like phenomenon in the RDM task. We first implemented the conditions to conduct an experiment with a so-called ‘double receptor design’ (Mori and Kai, 2002; Kitajo et al., 2003). We modified the montage of the first RDM apparatus by using two monitors, separated by a black divider centered at the midline of the viewing field. We applied the noisy signal to one eye, and the stimulus signal to the other eye, excluding the possibility of peripheral SR elicited in the eyes or retinas (‘optimized’ stimulus parameters: coherence = 5%; luminance = 25%; **Figure 4A**; see Materials and Methods). This approximation guaranteed that if the choices of the volunteers still exhibited SR under these conditions, then it would be centrally produced, in relays located after the optic chiasm, presumably in the primary visual cortex where the noise and stimulus signals first converge (Mori and Kai, 2002).

We performed experiments in 28 new subjects some of which received the visual stimulus to their left eye (*n* = 16) while others received it on their right eye (*n* = 12). We found similar %CCI peaks when providing the RDM stimulus to either the left (gray circles) or right (black circles) eye, indicating that the side of the eye that received stimulation did not affect the SR phenomenon (**Figure 4B**). We averaged the %CCI curves associated with the RDM projected to both eyes to obtain a global signature of the SR phenomenon using the double receptor design (**Figure 4C**). The resulting averaged %CCI curve had a peak of 5.3 ± 1.6% at a noise luminance of 5% (paired *t*-test, *P* < 0.0001) but an %CCI ≈1 when using 25% noise luminance. We quantified the amount of ‘inter-ocular SR’ by taking the ratio of the peak from the averaged %CCI curve obtained with the double receptor design (**Figure 4C**) to the one we observed with the single receptor design (**Figure 3C**). The amount of ‘inter-ocular SR’ was 70%, indicating that this phenomenon had monocular and binocular components, yet the latter was considerably larger (Ball and Sekuler, 1987). Therefore, the SR-like phenomenon in the RDM task mainly occurred at (or after) a stage in the visual system where binocular inputs interact (i.e., a stage higher than layer IV of the primary visual cortex Schoups and Orban, 1996).

### Variable Noise Sensitivity across Subjects

**FIGURE 5:**
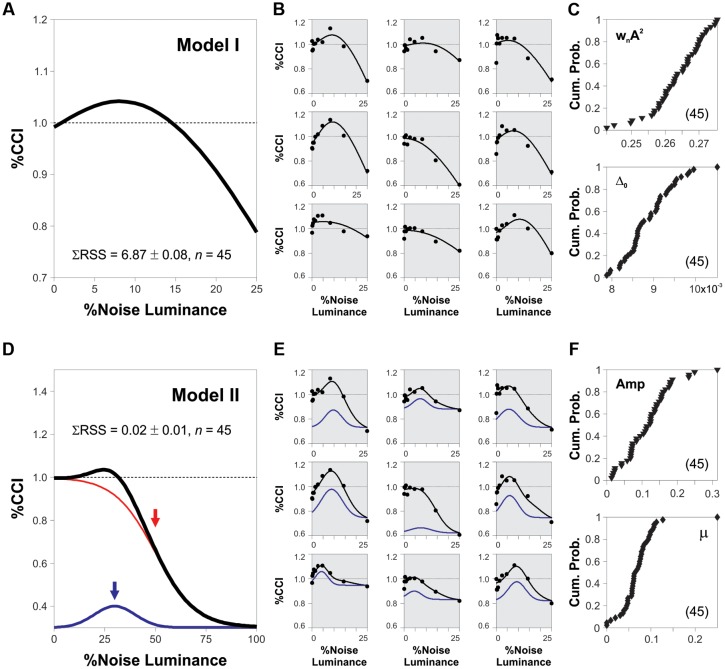
**Heterogeneity in SR properties across subjects.** **(A,D)** Synthetic %CCI curves as a function of background noise created with either model 1 or model 2, respectively (see Materials and Methods). **(B,E)** Fits of models 1 and 2 to the %CCI data from the same nine sample subjects (shaded panels). **(C,F)** Cumulative probability distributions for the fitted parameters to data from different subjects (see Materials and Methods). Low correlations between these parameters: 0.11 for model 1 **(C)** and 0.06 for model 2 **(F)**.

Our results demonstrate that the peak amplitude of the averaged SR curves depends on both the coherence of the RDM stimulus and the luminance of the background noise. Does the %CCI data from subjects contain individual differences in their corresponding measures for noise luminance and %CCI peaks? We compared our experimental results with the theory of SR by using an equation that describes the SR phenomenon itself. We did these adjustments exclusively to extract and compare SR-dependent measures across subjects. Thus, we adapted a model proposed by Moss et al. (2004; model 1; see Materials and Methods) and made non-linear fits to the %CCI data from 45 subjects that were involved in the experiments described and illustrated in **Figures 2** and **3**. We show the non-linear fits to nine sample cases in **Figure 5B** (shaded panels). From these analyses, we took the optimized parameters from each subject and constructed the cumulative probability distributions for the obtained fits for w_n_A^2^ (proportional to the peak of the %CCI curve) and Δ_0_ (proportional to the luminance at which the %CCI curve peaked; see Materials and Methods for more details). These two parameters spanned over a broad range of values and displayed a variance of normalized indexes of 5.6 and 4.9%, respectively, revealing strong differences in the SR-like phenomenon across subjects. We included a second descriptive model to extract direct measures for the peak amplitude and noise luminance linked to the %CCI peak from each subject (see Materials and Methods). We made a non-linear fit (black trace) that involved the sum of a Gaussian distribution (blue trace) with a logistic function (red trace; **Figure 5D**). From these fits, we extracted the luminance peaks and %CCI amplitudes for all the subjects. In **Figure 5E**, we illustrate the non-linear fits to data from nine sample subjects (shaded panels; same data as in **Figure 5B**). Although, these fits produced smaller errors (Kruskal–Wallis *F*_2,86_ = 66.75, *P* < 0.001), they still presented a broad range of peak values and optimal noise luminance, confirming the strong diversity in the underlying %CCI curves (variance of normalized indexes of 4.9 and 2.5%, respectively; **Figure 5F**). Notably, the %CCI peak amplitudes (**Figure 5F**, upper panel) and mean noise luminances at peak (**Figure 5F**, lower panel) were poorly correlated (*R^2^* = 0.06, *n* = 45). These results indicate that the %CCI curves exhibited different profiles, with strong diversity among individuals. The existence of small peak %CCI amplitudes reveals that at least some subjects did not display strong SR-like effects.

Finally, we tested the contribution of 19 individual characteristics such as age, sex, and years of education (among other factors; see **Table 1**) on the estimated peak amplitude and noise luminance at peak %CCI derived from the second predictive model (see Materials and Methods). We asked whether any of these characteristics from the subjects could predict properties from their %CCI curves. We indexed and grouped these properties by using a median split of the amplitude at luminance (**Table 1A**) or the noise luminance at peak (**Table 1B**) extracted from each subject and made statistical comparisons between ‘lower’ and ‘higher’ performers (Treviño, 2014). We found no differences between groups indicating that none of the individual factors that we characterized predicted the SR-like phenomenon in the RDM task (**Table 1**).

**Table 1:** Contribution of individual characteristics to the SR-like phenomenon.

	(A) Peak SNR amplitude				(B) Noise lum. at peak			
		
	‘Lower’	‘Higher’			‘Lower’	‘Higher’
								
	Mean ± SEM	Mean ± SEM	*F*	*P*	Mean ± SEM	Mean ± SEM	*F*	*P*
Age (years)	23.83 ± 1.00	22.41 ± 1.03	1.06	0.30	24.22 ± 1.00	22.00 ± 1.03	2.97	0.08
Sex	1.61 ± 0.11	1.50 ± 0.11	0.53	0.47	1.61 ± 0.11	1.50 ± 0.11	0.53	0.47
Height	166.65 ± 1.64	168.91 ± 1.68	0.83	0.36	166.30 ± 1.87	169.27 ± 1.92	1.17	0.28
Weight	65.35 ± 1.89	67.50 ± 1.93	0.18	0.67	64.30 ± 1.72	68.59 ± 1.76	1.14	0.28
Laterality	1.22 ± 0.11	1.05 ± 0.11	1.89	0.17	1.17 ± 0.10	1.09 ± 0.11	0.21	0.65
Glasses	0.52 ± 0.11	0.41 ± 0.11	0.56	0.45	0.52 ± 0.11	0.41 ± 0.11	0.56	0.45
Hours of sleep	6.26 ± 0.35	5.55 ± 0.36	3.10	0.08	5.93 ± 0.35	5.89 ± 0.36	0.00	0.95
Hours awake	5.00 ± 0.67	5.18 ± 0.68	0.17	0.68	4.41 ± 0.52	5.80 ± 0.54	2.23	0.14
Food	2.52 ± 0.20	2.55 ± 0.21	0.01	0.94	2.30 ± 0.21	2.77 ± 0.21	2.28	0.13
Portions	2.09 ± 0.21	1.86 ± 0.22	0.42	0.52	2.17 ± 0.21	1.77 ± 0.21	1.88	0.17
Time since last meal	6.04 ± 0.90	4.59 ± 0.92	1.20	0.27	6.46 ± 0.91	4.16 ± 0.94	3.12	0.08
Drink	0.39 ± 0.11	0.55 ± 0.11	1.05	0.31	0.39 ± 0.11	0.55 ± 0.11	1.05	0.31
Type of drink	0.65 ± 0.25	1.36 ± 0.25	1.62	0.20	0.78 ± 0.31	1.23 ± 0.32	1.35	0.25
Quantity of drink	0.52 ± 0.16	0.68 ± 0.16	0.64	0.42	0.48 ± 0.14	0.73 ± 0.15	1.11	0.29
Career	2.43 ± 0.13	2.18 ± 0.13	1.33	0.25	2.30 ± 0.15	2.32 ± 0.15	0.00	1.00
Degree	3.48 ± 0.16	3.55 ± 0.16	0.04	0.84	3.57 ± 0.16	3.45 ± 0.16	0.79	0.37
Grades	87.52 ± 1.26	80.77 ± 1.29	0.00	0.99	87.39 ± 1.22	80.91 ± 1.25	0.00	1.00
Work	0.48 ± 0.11	0.36 ± 0.11	0.59	0.44	0.48 ± 0.11	0.36 ± 0.11	0.59	0.44
Video games	0.22 ± 0.09	0.23 ± 0.09	0.01	0.94	0.13 ± 0.07	0.32 ± 0.08	2.24	0.13


*We tested the influence of some individual characteristics (arraged in rows) on the the peak %CCI amplitude **(A)** or the noise luminance at peak **(B)** which values were extracted from the data fits to the second predictive SR model (see Materials and Methods). No differences between ‘lower’ and ‘higher’ performers identified by a median split of each dataset indicating that none of these individual factors predicted the SR-like phenomenon in the RDM task.*

## Discussion

We adapted a RDM task (Newsome and Pare, 1988; Gold and Shadlen, 2007) where the subjects had to choose between two known opposite directions of motion (Kiani and Shadlen, 2009). Choice performance in this task improves with viewing duration, implying that information for each option accumulates over time (Gold and Shadlen, 2007; but see Zariwala et al., 2013). The rate of this accumulation process depended on both the coherence and luminance levels of the moving dots (Gold and Shadlen, 2007). Hence, when plotted as a function of %correct choices, the RTs followed an inverted bell-shaped pattern with smaller RTs for ‘easy’ and ‘difficult’ tasks, revealing the so-called ‘speed-accuracy tradeoff.’ This response pattern is probably one of the most frequently replicated findings in experimental psychology (Smith and Ratcliff, 2004).

Once we ensured the steady-state of our psychophysical measurements, we asked how adding background pixel-noise with different luminance levels influenced the perception of coherent motion in the RDM task. In linear systems, the addition of noise to either the system or the input stimulus degrades signal quality (Lugo et al., 2008). In non-linear systems, however, the addition of an intermediate level of noise can enhance signal detection and transmission (Harper, 1979). Here, we found that adding background pixel-noise with 5% luminance increased the %correct choice index (%CCI, see Materials and Methods) of the RDM task by 7% when conducted under low coherence conditions (5–8%). In one set of experiments, we allowed the subjects to control their viewing and response times autonomously. However, it is well-known that the shape of the RT distribution and response accuracy co-vary as a function of the experimental condition (Smith and Ratcliff, 2004). We thus conducted a second round of experiments in which we fixed the stimulus presentation intervals to 1 s. For both conditions, we found that the %CCI curves followed an inverted U-like pattern as a function of background noise levels. The %CCI was first enhanced by the noise, up to a maximum, and then lessened. The %CCI enhancement was insufficient in the case of little noise, whereas too high noise degraded discrimination (Moss et al., 2004; Jung and Marchesoni, 2011). It was an optimal amount of noise with 5% luminance which maximized the %CCI curves (Zeng et al., 2000; Sasaki et al., 2006) suggesting the occurrence of a SR-like phenomenon. To our knowledge, this is the first study that shows the beneficial effect of white noise at the discrimination level in a visual task (rather than at the detection level).

We also compared the RTs from trials performed under different noise luminance conditions, but found no differences across them. This implies that the increase in motion discrimination performance produced by visual noise cannot be explained by augmented RTs. Interestingly, the averaged %CCI curve extracted from error trials that had a RT below the median was insensitive to visual noise. Thus, the SR-like effect in the RDM task is a visual perceptual phenomenon. An alternative explanation for the observed peaks in the %CCI curves is that small amounts of noise could also reduce the observer’s uncertainty to solve the task (Pelli, 1985; Blackwell, 1998). In the uncertainty model, an observer monitors many channels of information from which only a subset is relevant for the discrimination task. Thus, noise could directly increase the activity of the relevant channels, or reduce the irrelevant ones, resulting in a decrease in the uncertainty of some relevant aspect of the signal (Pelli, 1985). It’s important to keep in mind that in SR, subthreshold summation can take place only when the noise overlaps with spectral bands of the signal, whereas uncertainty reduction can occur without this requirement.

A common view is that choosing the right option in the RDM task involves some form of statistical inference (Gold and Shadlen, 2007). This leads to a quantitative link between the time-course of a behavioral decision, the growth of stimulus information and the correct choice probability (Smith and Ratcliff, 2004). Our results suggest that visual noise can play a constructive role at the discrimination stage. Therefore, the presence of small amounts of background noise in the visual system might increase the performance in motion discrimination and thereby influence choice behavior (Ward et al., 2002).

Stochastic resonance exists across a wide variety of experimental conditions. It has been found to improve sensory detection in audition (Zeng et al., 2000), vision (Simonotto et al., 1997), and touch (Wells et al., 2005). The SR phenomenon is present when applying signal and noise to the same (Riani and Simonotto, 1994; Collins et al., 1996; Levin and Miller, 1996; Sasaki et al., 2006) or to different sensory modalities (Martinez et al., 2007). Although light activates visual photoreceptors, the direction-selective neurons involved in visual motion perception in the RDM task belong to higher visual system circuitry and locate in the middle temporal area (MT; Maunsell and Van Essen, 1983). Newsome et al. (1989) demonstrated the functional role of these neurons from the dorsal pathway in an interesting study in which chemical lesions of this region in monkeys impaired their behavioral performance on the direction discrimination task (Newsome and Pare, 1988). Consistently with this finding, it was later shown that electrical micro-stimulation of direction-selective neurons in the MT of the cerebral cortex of monkeys and humans influenced their perceptual judgment of motion direction (Salzman et al., 1992). Our results reveal that external visual noise can also improve motion perception in the same visual task. Also, a recent study shows that the application of transcranial random noise electrical stimulation (tRNS) of the occipital region in humans enhances visual perception of static visual stimuli (van der Groen and Wenderoth, 2016). What are the exact cellular and circuit-level mechanisms involved in the SR-mediated improvement of discrimination tasks? At the cellular level, SR has been observed in diverse models of spiking neurons (Bulsara et al., 1991; Ward et al., 2002). Due to the non-linearity in the firing threshold of the neural cells, applying intermediate noise levels to a sub-threshold input signal will increase, through a SR phenomenon, the number of spikes produced by the cell (Zeng et al., 2000; Moss et al., 2004; Martinez et al., 2007). Currently, we are investigating the cellular mechanisms involved in SR by using optogenetic noise photo-stimulation (Manjarrez et al., 2015). Indeed, with the optogenetic tools, we can specifically increase the noise sources into pyramidal cells in the binocular region of the mouse visual cortex. This approximation might provide valuable new insights into the underlying circuit level mechanisms involved in SR and decision-making.

Although, we found that the effects of background noise in the RDM task were highly reproducible and robust, there were strong individual differences in the %CCI curves implying that some levels of noise were beneficial for some subjects but detrimental for others. What could predict these differences across subjects? One plausible explanation is that electrochemical noise generated internally in the brain (internal noise), combines with the noise added by the experimenter (external noise) to determine the net amount of noise mixed with the input signal (Mori and Kai, 2002; Ward et al., 2002). The variability in %CCI curves could derive from individual differences in attention (Prince, 2008), cognition (Braver and Barch, 2002), or other motivational factors (Wise, 2004; Grace et al., 2007). Thus, although we partially controlled the external noise sources, the internal noise was not under any control and probably varied across trials, subjects and replications (Ward et al., 2002). An interesting possibility would be that the internal noise level determined how the external visual noise enhanced the discriminability of the weak RDM signal (Aihara et al., 2008).

Derived from the analysis we did on the %CCI curves from individuals, we also found that the peak amplitudes and the noise luminance at which those curves reached their peaks were not correlated with each other. Contrast modulation of psychophysical responses is mediated by a complex cascade of cellular and synaptic interactions that starts with retinal ganglion cells (Enroth-Cugell and Robson, 1966; Baden et al., 2016), and percolates to thalamic (Derrington and Lennie, 1984) and visual cortical (Movshon et al., 1978; Albrecht and Hamilton, 1982; Glickfeld et al., 2013) circuits. Neurons from all these circuits operate as high-pass filters for contrast and encode a broad range of ‘classical’ features such as spatial frequency and orientation (Albrecht and Hamilton, 1982). Yet, interestingly, these encoded properties are unaffected by changes in contrast (Carandini and Ferster, 2000). Maybe this could explain why the noise luminance poorly predicted the peak of the %CCI curves in the RDM task.

In our main experiments, we adopted a ‘single receptor design’ by delivering signal and noise simultaneously to both eyes. We exploited the fact that the eyes are cross-wired to the visual cortex, so we bypassed the peripheral sites and tested for the phenomenon directly in the human visual cortex. Using a ‘double receptor design,’ we delivered the signal and noise separately to each eye, forcing these two elements to converge first at central synapses in the brain (Mori and Kai, 2002). We reasoned that if the visual noise affected neurons located in monocular regions, then the noise-enhancing effects should be absent when presenting the RDM stimulus to the pathway responding to the other eye. That would be consistent with a SR-like phenomenon acting at or before area 17, where the binocular cells first appear (Ball and Sekuler, 1987). However, our results revealed a non-retinotopic phenomenon in higher brain areas that engage in sensory processing from both eyes (Rodriguez et al., 1999). Indeed, the subjects showed considerable ‘inter-ocular SR’ and therefore, a significant portion of the SR phenomenon was localized either at or after the primary visual cortex layer IV (Schoups and Orban, 1996). We propose that it was mainly in cortical circuits where noise and signal had the relevant interactions that produced the SR-like phenomenon in the RDM task (Kitajo et al., 2003). Although the exact mechanisms for binocular interaction remain unclear, these results might contribute to understanding how the brain combines information from both eyes (Kitajo et al., 2003).

Noise can improve the SNR in physical and biological systems. The existence of SR phenomena in neural systems raises great interest because it can increase the detectability of relevant stimuli. Some authors suggest that the central nervous system might utilize noise, and even tune their endogenous noise levels, to enhance sensory information (Moss et al., 2004). We observed SR-like behavior in a discrimination task both at the individual and group levels under different experimental conditions. The repeatability and stability of our results suggest that SR might play a major role in the way the human visual system processes sensory stimuli. Moreover, it could be potentially used to refine the perceptual processing of suboptimal stimuli and maybe also to repair some discrimination deficiencies (Collins et al., 1996). Because multiple brain disorders and pathologies consist of a reduced firing rate of sensory neurons, the SR could be employed to develop methods for enhancing human discriminative performance in non-invasive ways (Collins et al., 1996). The fact that SR occurs at a macroscopic scale in human behavior opens the way to many applicative consequences involving the enhancement of natural and artificial sensation (Medina et al., 2012).

## Author Contributions

MT: designed and performed experiments, analyzed data, made figures, drafted and wrote the manuscript; BT-V: analyzed data; EM: designed experiments and wrote the manuscript. All authors discussed and approved the final version of the manuscript.

## Conflict of Interest Statement

The authors declare that the research was conducted in the absence of any commercial or financial relationships that could be construed as a potential conflict of interest.
